# Transdiagnostic Symptom Subtypes to Predict Response to Therapeutic Transcranial Magnetic Stimulation in Major Depressive Disorder and Posttraumatic Stress Disorder

**DOI:** 10.3390/jpm12020224

**Published:** 2022-02-06

**Authors:** Camila Cosmo, Yosef A. Berlow, Katherine A. Grisanzio, Scott L. Fleming, Abdullah P. Rashed Ahmed, McKenna C. Brennan, Linda L. Carpenter, Noah S. Philip

**Affiliations:** 1 Department of Psychiatry and Human Behavior, The Warren Alpert Medical School, Brown University, Providence, RI 02912, USA; camila_cosmo@brown.edu (C.C.); yosef_berlow@brown.edu (Y.A.B.); Linda_Carpenter_MD@brown.edu (L.L.C.); 2 VA RR&D Center for Neurorestoration and Neurotechnology, VA Providence Healthcare System, Providence, RI 02908, USA; mckenna.brennan@va.gov; 3 Center for Brain Science, Department of Psychology, Harvard University, Cambridge, MA 02138, USA; kgrisanzio@g.harvard.edu; 4 Department of Biomedical Data Science, Stanford University, Stanford, CA 94305, USA; scottyf@stanford.edu; 5 Department of Neuroscience, Brown University, Providence, RI 02912, USA; abdullah_rashed_ahmed@brown.edu; 6 COBRE Center for Neuromodulation, Butler Hospital, Providence RI 02906, USA; 7 Butler Hospital TMS Clinic and Neuromodulation Research Facility, Providence, RI 02906, USA

**Keywords:** transdiagnostic, symptom subtypes, anxious arousal, TMS, biomarker, linear discriminant analysis

## Abstract

The diagnostic categories in psychiatry often encompass heterogeneous symptom profiles associated with differences in the underlying etiology, pathogenesis and prognosis. Prior work demonstrated that some of this heterogeneity can be quantified though dimensional analysis of the Depression Anxiety Stress Scale (DASS), yielding unique transdiagnostic symptom subtypes. This study investigated whether classifying patients according to these symptom profiles would have prognostic value for the treatment response to therapeutic transcranial magnetic stimulation (TMS) in comorbid major depressive disorder (MDD) and posttraumatic stress disorder (PTSD). A linear discriminant model was constructed using a simulation dataset to classify 35 participants into one of the following six pre-defined symptom profiles: Normative Mood, Tension, Anxious Arousal, Generalized Anxiety, Anhedonia and Melancholia. Clinical outcomes with TMS across MDD and PTSD were assessed. All six symptom profiles were present. After TMS, participants with anxious arousal were less likely to achieve MDD remission compared to other subtypes (FET, odds ratio 0.16, *p* = 0.034), exhibited poorer PTSD symptom reduction (21% vs. 46%; t (33) = 2.025, *p* = 0.051) and were less likely to complete TMS (FET, odds ratio 0.066, *p* = 0.011). These results offer preliminary evidence that classifying individuals according to these transdiagnostic symptom profiles may offer a simple method to inform TMS treatment decisions.

## 1. Introduction

Over the last decade, an expanded understanding of the complexity of mental health disorders has led to new approaches of psychopathological phenomenology [1]. The National Institute of Mental Health (NIMH) created the Research Domain Criteria (RDoC), a research initiative addressing mental health and its disorders based on functional dimensions investigated by many approaches, ranging from molecular to behavioral strategies [2,3]. In line with this initiative, the transdiagnostic approach has emerged as a new and promising paradigm for understanding psychopathologies through symptoms measured across classic psychiatric diagnoses [4,5].

As one example of this RDoC approach, Grisanzio et al. used a dimensional analysis of symptom ratings to identify unique transdiagnostic subtypes across 420 individuals with posttraumatic stress disorder (PTSD), major depressive disorder (MDD), panic disorder, and healthy control participants [4]. They identified six unique symptom clusters that were classified as Tension, Anxious Arousal, Generalized Anxiety, Anhedonia, Melancholia, and Normative Mood. These transdiagnostic symptom subtypes were shown to be represented across the psychiatric diagnoses, as well as in healthy controls. Furthermore, these symptom profiles were associated with differential performance on precision measures of neurocognition, neurophysiological measures of brain activation, and reporting of daily functional capacity. The authors demonstrated the reproducibility of these symptom subtypes through replication in an independent sample [4]. However, it is unclear whether these transdiagnostic symptom clusters could be used to inform and guide clinical treatment decisions. 

Considering that neuropsychiatric disorders are the leading cause of disability worldwide [6,7], and given the limited effectiveness of available pharmacological treatments for mood, anxiety, and trauma disorders [8,9,10,11], neuromodulation techniques, such as therapeutic transcranial magnetic stimulation (TMS), have been widely investigated [12,13,14,15,16,17,18]. While a comprehensive review of TMS is beyond the scope of this paper (e.g., see [17]), TMS uses rapidly fluctuating magnetic fields, titrated to an individual’s cortical excitability, to induce depolarization in targeted brain areas [19]. Therapeutic effects are linked to neural changes that occur both in the targeted area and in connected brain regions, particularly those involved in the default mode and other large-scale neural networks [20]. Recent neuroimaging work suggests that specific symptom clusters, derived from standard rating scales, may align with underlying neural circuitry, and are related to symptom response in depressed individuals [21,22]. At present, TMS treatments are administered on a daily basis for up to six weeks, although novel approaches, such as sessions with reduced administration time [23] or greater cumulative TMS doses over a shorter period of time [24], are emerging as promising new ways to deliver therapeutic TMS.

With these considerations in mind, therapeutic TMS (regardless of how it is delivered) carries a significant burden of cost and time commitment to the patient and provider. To this end, inexpensive predictive markers are needed to identify patients most likely to respond to TMS. If this approach is successful, it will yield a low-cost, easily implemented approach that is well suited to clinical TMS settings. We, thus, investigated whether the pre-identified symptom clusters described by Grisanzio et al. [4] would have prognostic value as predictors of therapeutic response to TMS. To achieve this goal, we performed a secondary analysis of data from adults with comorbid MDD and PTSD, since this comorbidity is clinically common and the sample can be considered enriched with regard to the transdiagnostic symptoms of depression, PTSD and anxiety utilized for symptom cluster identification [6]. We hypothesized that the pre-identified symptom clusters would be present and predictive of clinical response in this sample; determination of which symptom clusters would be most relevant to outcomes in specific MDD or PTSD domains was exploratory. 

## 2. Materials and Methods

### 2.1. Trial Design

The parent prospective unblinded multi-site trial was performed at Butler Hospital and the Providence Veterans Affairs Medical Center. Study details are provided below; for comprehensive information regarding the methods and outcomes of the original trial, see Carpenter et al.’s paper [14].

### 2.2. Ethics Statement

The Providence VA and Butler Hospital Institutional Review Boards approved the study protocol. Following the ethical principles of the Declaration of Helsinki for clinical research, all individuals were provided with detailed verbal and written information about the study and provided written informed consent for participation in the trial (ClinicalTrials.gov NCT02273063).

### 2.3. Participants

Thirty-five individuals participated in the parent trial based on the following inclusion criteria: (a) diagnosis of PTSD and MDD based on DSM-IV criteria (verified by board-certified psychiatrists NSP and LLC with extensive experience treating patients with these disorders); (b) presented illness severity rating of at least “moderately ill”, for both disorders, on the Clinical Global Impressions Severity Scale (CGI-S); (c) between the ages of 18 and 75 years; (d) failure of at least one evidence-based antidepressant trial; (e) had a prior stable psychotropic regimen for at least six weeks before enrollment.

Individuals were excluded from the study if they (a) had prior rTMS treatment; (b) had any primary psychotic disorder, bipolar I disorder, or ongoing substance use disorder; (c) were actively suicidal; (d) had any other TMS-specific exclusion criteria, such as pregnancy risk, history of moderate or severe traumatic brain injury, active unstable medical conditions, or severe neurological disorders/impairment, including CNS tumors, seizure disorders, or cerebrovascular disease.

### 2.4. Assessments

The parent study included the following rating scales: (a) the Inventory of Depressive Symptomology—Self Report (IDS-SR) for measuring MDD severity; (b) the PTSD Checklist for DSM-5 (PCL-5) to assess PTSD symptoms; (c) the 42-item Depression Anxiety Stress Scale (DASS). IDS-SR and PCL-5 scores were collected at baseline, biweekly during the first 4 weeks, weekly during the remainder of treatment, and within 72 h after the final treatment session. DASS scores were collected at baseline and post treatment. Clinical rating scale data from all 35 adult subjects, included in the parent trial, were used in this study (i.e., the entire intent-to-treat sample) [14]. 

### 2.5. TMS Parameters

Participants received up to 40 rTMS sessions, once daily, for 7 consecutive weeks (on business days), with the last 5 sessions delivered on a taper schedule over 3 weeks. Stimulation was delivered with a figure-8 coil at 5 Hz to the left dorsolateral prefrontal cortex (DLPFC), targeted via the Beam/F3 method [25], at 120% of motor threshold, with sessions of 3000 pulses, using the NeuroStar TMS Therapy System (Neuronetics, Inc., Malvern, PA, USA) (see Carpenter et al. [14] and Philip et al. [26] for further details and description of the rationale behind TMS parameter selection).

### 2.6. Safety

Safety assessment was performed at the end of each rTMS session through the documentation of spontaneously reported side effects. These events were categorized as serious or nonserious.

### 2.7. Statistical Analysis

**Figure 1:**
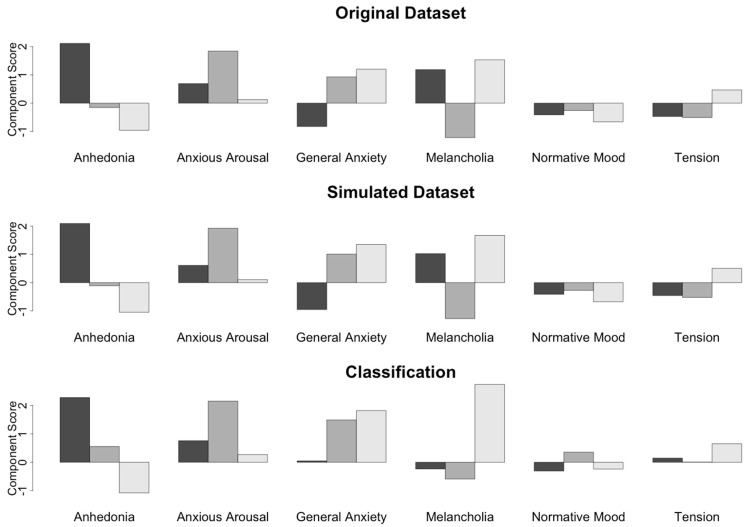
Linear discriminant model to classify subjects. The original dataset (top) was simulated (middle) using a sample of normally distributed component scores with the same means and standard deviations as the six symptom profile subtypes identified in the original dataset. A linear discriminant model based on these profiles was then constructed and used to classify subjects based on their symptom component scores (bottom).

Using item-level data from baseline DASS scores, three primary components, labeled anhedonia, anxious arousal and tension, were calculated for each participant based on the principal component analysis, scaling, and item loadings described by Grisanzio et al. [4]. The anhedonia component was loaded with statements such as “I felt that life was meaningless,” “I found that I had nothing to look forward to,” and “I couldn’t experience any positive feelings at all.” The anxious arousal component loading included items such as “I felt close to panic,” “I felt scared without any good reason,” and “I was aware of the action of my heart in absence of physical exertion.” The tension component included statements such as “I felt I was rather touchy,” “I found it hard to wind down,” and “I found it difficult to relax.” [4]. A simulated dataset was created to approximate the distributions of these primary components across subtype clusters as they were reported in the original dataset [4]. Simulated subtype profiles with n’s equal to the published sample sizes were created using normally distributed points that matched the published means and standard deviations of the component z-scores for each cluster (Figure 1). This simulation method provided an approach to apply the original subtype profiles to a new sample without the full original dataset. A linear discriminant analysis (LDA) model was then constructed using this simulated dataset implemented using the MASS package in R [27]. This LDA model was then used to classify subjects into one of six pre-defined symptom profiles based on the similarity of the three calculated component scores. The profiles included the following: normative mood, tension, anxious arousal, generalized anxiety, anhedonia and melancholia (as illustrated in Figure 1). Rates of remission, defined as an IDS-SR score less than or equal to 14 at last observation, were then assessed across all profile subtypes using the Fisher’s exact test (FET). Based on this initial analysis, a binary grouping of subtypes was identified. Additional treatment outcomes, including percent change in depression ratings and PTSD symptoms and rates of completion of the TMS treatment protocol, were then compared using FET for categorical data and t-tests for continuous data. Odds ratios and the corresponding 95% confidence intervals (CI) for FET were calculated using minimum likelihood [28]. Sensitivity, specificity and accuracy of baseline grouping in relation to depression remission rates were calculated. All analyses were conducted in R [29].

## 3. Results

This sample included 14 women and 21 men, aged 27 to 67 years. Sixty percent of these participants had previous psychiatric inpatient hospitalization. The depression scores, as measured by the IDS-SR total scores, significantly decreased from baseline (mean ± SD, 47.8 ± 11.9) to post treatment (30.9 ± 18.9; t (34) = 6.36, *p* < 0.001). See Carpenter et al. [14] for demographic and clinical characteristics and outcomes, and Philip et al. [26] for associated neuroimaging results.

The linear discriminant model identified all six symptom profiles in the sample in the following distribution: anxious arousal (43%), anhedonia (20%), tension (20%), normative mood (9%), general anxiety (6%), and melancholia (3%). As expected, the baseline total DASS scores varied by subtype with general anxiety at 86.0 ± 11.3, anxious arousal at 84.3 ± 20.8, anhedonia at 67.6 ± 16.2, melancholia at 64 ± NA, tension at 43.9 ± 18.9, and normative mood at 26.7 ± 15.7. Similarly, the mean baseline depression ratings, as measured by IDS-SR scores, also varied by subtype, with anxious arousal at 54 ± 10, anhedonia at 50 ± 10, melancholia at 49 ± NA, general anxiety at 49 ± 2.0, tension at 37 ± 10, and normative mood at 32 ± 6.2. 

**Figure 2:**
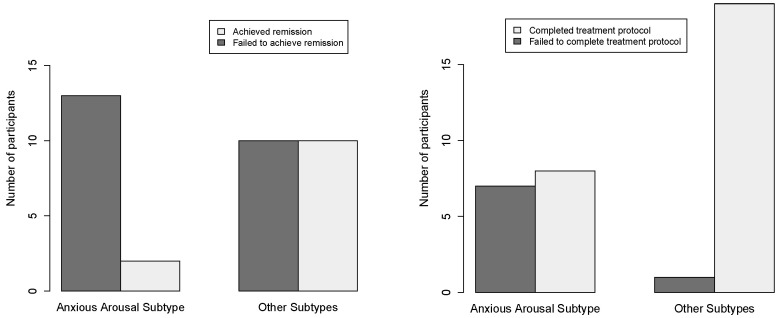
Left: After rTMS compared to all other subjects (FET, odds ratio 0.16, *p* = 0.034). This grouping predicted successful remitters with a sensitivity of 0.83, specificity of 0.56 and accuracy of 66%. Right: subjects in the anxious arousal subtype were also less likely to complete the treatment protocol (FET, odds ratio 0.066, *p* = 0.011).

The post-treatment depression remission rates differed significantly across the subtypes (FET, *p* = 0.047), with anxious arousal exhibiting the lowest remission rate (13%, n = 2/15) (Table 1). When compared to all the other subtypes, participants classified in the anxious arousal subtype were less likely to achieve MDD remission (FET, odds ratio 0.16, 95% CI: 0.021–0.92, *p* = 0.034). This difference corresponded with smaller percent reductions in IDS-SR scores after TMS in the anxious arousal group compared to other participants (24 (±34)% vs. 48 (±27)%, t (33) = 2.36, *p* = 0.024). This binary grouping at baseline identified successful remitters with a sensitivity of 0.83, specificity of 0.56 and accuracy of 66%. The subjects in the anxious arousal subtype also demonstrated a nominally smaller reduction in PCL-5 after TMS compared to all the other subtypes (21 (±38)% vs. 46 (±35)%; t (33) =2.025, *p* = 0.051), and were less likely to complete the treatment series (FET, odds ratio 0.066, 95% CI: 0.0026–0.53, *p* = 0.011) (Figure 2). The anxious arousal component score appeared to drive this effect, and a simple model using a cutoff score of 1.0 for this standardized component score at baseline yielded reasonable separation between subjects who later met the post-treatment remission criteria and those who did not, correctly classifying 74% of the sample with a sensitivity of 0.59 and specificity of 0.89.

## 4. Discussion

This study assessed the application of transdiagnostic symptom clusters as described by Grisanzio et al. [4] as potential predictors of TMS response in adults with MDD and comorbid PTSD. Linear discriminant analysis was used to classify individuals according to cluster subtype (Tension, Anxious Arousal, Generalized Anxiety, Anhedonia, Melancholia, or Normative Mood), and the relationships between these subtypes and TMS treatment outcomes were assessed. Anxious Arousal was the predominant symptom cluster (43%) in our sample. This subtype was found to have the lowest depression (IDS-SR) remission rate (13%) and a smaller reduction in PTSD (PCL-5) symptoms (21%) following 5 Hz TMS treatment compared to the other subtypes. Our findings indicate the potential of applying these symptom profile subtypes as predictors of response and remission in individuals with MDD and PTSD who are receiving 5 Hz TMS treatment using standard targeting methods. 

In the original study that proposed these six symptom clusters across MDD, PTSD, and panic disorder, the anxious arousal subtype was the most prevalent subtype (26%, n = 53/200), after excluding healthy controls [4]. This cluster had the worst behavioral performance on neurocognitive tests among all the subgroups, reflecting poor inhibitory control and working memory. In addition, this subtype was marked by a poor daily functional capacity, demonstrated through deficits in social skills and low emotional resilience. Our findings of a lower remission rate and decreased response among individuals in the anxious arousal cluster seem consistent with Grisanzio et al. [4]. In other words, patients displaying anxious arousal features appear less likely to respond to TMS when compared to the other symptom clusters. Of note, this cluster was associated with greater cognitive impairment and reduced functional capacity, indicating that these may be areas of inquiry for future symptom-based predictors of response.

Aligned with this transdiagnostic approach, and based on the premise that PTSD and depression share cardinal symptoms, Contractor et al. examined depression and PTSD symptom constructs based on PTSD diagnosis status [30]. The authors observed that the PTSD anxious arousal cluster was more distinct in its association with non-somatic depression, whereas dysphoric arousal was prominently related to somatic depressive symptoms [30]. The anxious arousal cluster was also found to be linked to alcohol use disorder in veterans with PTSD in multivariable logistic regression and relative importance analyses performed by Palmisano et al. [31]. Interestingly, the association continued to be significant, even after controlling for depression. Although these studies have not specifically investigated transdiagnostic subtypes, as was performed by Grisanzio et al. [4] and by our study, they reiterate the importance of conceptualizing and examining symptom clusters, instead of applying disorder-specific approaches [32], and they have potential to lead to rationally designed personalized therapeutic approaches, in addition to informing the development of markers of response. 

Neuromodulation techniques, such as TMS, have advanced significantly in the last two decades, with promising outcomes in clinical and research settings. So far, the US Food and Drug Administration has cleared devices to deliver TMS as an evidence-based treatment for pharmacoresistant MDD, obsessive-compulsive disorder, and as an adjunct to smoking cessation, with promising data for its use in PTSD (e.g., [15,33]) and suicidality (e.g., [34]). TMS has been widely investigated, targeting several neuropsychiatric symptoms and disorders [12,13,14,15,16,17,18,35] in trials using a variety of targets and stimulation protocols. Yet, one of the biggest challenges to further expanding its application is the lack of effective predictive markers that may identify individuals who are most likely to respond to TMS treatment, either in its commonly administered form or in the context of novel stimulation protocols. In addition to clarifying the underlying mechanisms of TMS, such markers would optimize TMS use and predict tailored treatments, resulting in a more efficient time and economic profile. In this context, studies based on neurophysiological and functional neuroimaging methods have examined and identified several TMS response biomarkers [36,37,38]. Nevertheless, these approaches are primarily high cost, technically complex, and time consuming, limiting their feasibility in the clinical setting. Therefore, as applied in this study, response markers based on self-reported symptom profiles may represent viable, simple, and inexpensive means of personalizing and guiding TMS treatment decisions, likely with a higher probability of translating them into clinical use.

There are several important limitations to this study. Foremost among these is the small sample size, which, while comparable to other rTMS trials, precludes more complex analysis and more robust conclusions regarding unique characteristics of the symptom profiles and the interactions of the symptom components. As the cluster subtypes are defined by symptom severity across three independent components, it is not surprising that the two subtypes defined by lower component scores, normative mood and tension, had relatively lower baseline depression scores compared to other subtypes, and this difference in baseline symptom severity likely contributed to the current findings. However, it is notable that the remaining four clusters had similar total depression ratings at baseline (IDS-SR scores ranged from 49 to 54), while demonstrating unique patterns of symptom severity on the DASS component scores and different rates of treatment remission. The low subject number only supported targeted analysis of the largest subtype, anxious arousal, which was defined by a high component score. Despite this limited exploration of profiles, this study provides initial support for the idea that the anxious arousal component is an important negative predictor of successful TMS treatment response. 

Additional limitations of this study include unblinded TMS administration, and, thus, the effect of the placebo response is likely relevant to the observed outcomes. Although all the participants were stable (>6 weeks) on medications, they were not medication free, and the nature of the interactions between other concurrent treatments and stimulation remains unknown, although the results are more consistent with everyday clinical practice. Furthermore, we did not assess symptom cluster profiles after the baseline assessment, so our results provide limited insight into whether these clusters might have additional utility to inform clinically relevant symptom profile changes over time, i.e., during or after a course of treatment. With these factors in mind, it is important to recognize that the current findings should be interpreted as preliminary until replicated in larger samples. That stated, the conceptual similarity between this and other areas of related inquiry, e.g., [22], indicates that this area is likely fruitful for future inquiry. Finding ways to definitively link the lessons learned from higher-cost neuroscience approaches to understanding TMS with low-cost symptom scales is an important area of future research in the field. 

## 5. Conclusions

This study offers preliminary evidence of the feasibility and utility of applying transdiagnostic symptom profiles to predict TMS treatment outcomes using linear discriminant analysis. Our results indicate that discrete subtypes are represented in patient populations of interest, and that the subtypes identified by Grisanzio et al. [4] are relevant to the response to treatment with TMS. Our results imply that classifying individuals according to these transdiagnostic symptom profiles may offer a simple and inexpensive method to help identify which patients might benefit most from a course of TMS treatment.

## Figures and Tables

**Table 1:** Baseline symptom clusters and remission rates after TMS.

	Symptom Profile Subtypes					
	Anhedonia	Anxious Arousal	Generalized Anxiety	Melancholia	Normative Mood	Tension
Achieved Remission	2	2	1	1	1	5
Failed to Achieve Remission	5	13	1	0	2	2

## Data Availability

The data presented in this study are available on request from the corresponding author. The data are not publicly available due to institutional restrictions.
